# Combined Bentall and Modified Ravitch Procedures: A Case Report and Systematic Review of the Evidence

**DOI:** 10.3390/medicina58121774

**Published:** 2022-11-30

**Authors:** Ali Taghizadeh Waghefi, Asen Petrov, Manuel Wilbring, Zuzana Fajfrova, Guido Fitze, Klaus Matschke, Utz Kappert

**Affiliations:** 1 Department of Cardiac Surgery, University Heart Center Dresden, 01307 Dresden, Germany; 2 Department of Pediatric Surgery, University Hospital Carl Gustav Carus, 01307 Dresden, Germany

**Keywords:** Ravitch, Bentall, Marfan syndrome, pectus excavatum

## Abstract

*Background and Objectives*: Marfan syndrome (MS) is a genetic disorder with autosomal dominant inheritance that affects the connective tissue and consequently many organ systems. The cardiovascular manifestations of MS are notorious and include aortic root dilatation or acute aortic dissection, which can cause morbidity and early mortality. However, surgical treatment of aortic pathology may be complicated by musculoskeletal deformity of the chest wall, as in pectus excavatum. In this regard, single-stage combined Bentall and Ravitch surgery is an extreme rarity that has also been scarcely reported in the literature. *Patients and Methods*: We present the medical history and single-stage Bentall and modified Ravitch surgical treatment of an 18-year-old male MS patient with symptomatic and severe pectus excavatum (PEX) in conjunction with a pear-shaped aortic root aneurysm. To discuss our case in the context of a synopsis of similar published cases, we present a systematic review of combined Bentall surgical aortic aneurysm repair and Ravitch correction of PEX. *Results*: A total of four studies (one case series and three case reports) and a case from our institution describing a single-stage combined Bentall and Ravitch operation were included. Patients were 22 ± 5.9 years of age (median = 22.5 years) and predominantly male (60%). All cases reported a midline vertical skin incision over the sternum. The most common surgical approach was midsternotomy (80%). In all cases metal struts were used to reinforce the corrected chest wall. Postoperative mortality was zero. *Conclusions*: Single-stage combined Bentall and Ravitch surgery is an underutilized surgical approach. Its use in MS patients with concomitant PEX and ascending aortic aneurysm that require surgical treatment warrants further investigation. Midsternotomy seems to be a viable access route that provides sufficient exposure in the single-stage surgical setting. Although operative time is long, the intraoperative and postoperative risks appear to be low and manageable.

## 1. Introduction

Marfan syndrome (MS) is a hereditary connective tissue disorder that encompasses multiple organ systems. Pathogenetic alteration in *FBN1,* which encodes the extracellular matrix glycoprotein fibrillin-1, has been identified as the cause of MS [1]. This multisystemic disease is transmitted in an autosomal dominant pattern and clinically characterized by age-related progression, pleiotropic manifestations, and a high degree of phenotypic variability. Clinical manifestations can be widespread but are particularly prominent in the musculoskeletal, ocular, and cardiovascular systems [2,3]. Hence, MS requires a multidisciplinary approach throughout the diagnostic and therapeutic phases. Musculoskeletal abnormalities, frequently the earliest signs of MS, play a fundamental role in assessment of patients suspected to have the disease [4]. Pectus excavatum (PEX), also known as funnel chest, is a common musculoskeletal deformity seen in two-thirds of MS patients [5] that is characterized by anterior chest wall depression into the thoracic cavity [6]. Inward deformities of the anterior chest wall, depending on their severity, may cause compression of the right heart as well as restrictive lung disease [7,8,9]. As a result, severe PEX may reduce exercise ability on its own [10]. Moreover, MS is also notorious for its cardiovascular manifestations, such as aortic root dilatation and acute aortic dissection, which are a serious cause of morbidity and early mortality [11,12]. Cardiosurgical treatment is often indispensable to treat these life-threatening conditions. However, such treatment is challenging in the presence of chest wall deformities such as PEX. To date, the number of publications reporting the various surgical strategies for combined PEX and aortic operations in MS patients is small, and most are case reports or case series. We report a single-stage combined Bentall and modified Ravitch operation in which an episternal steel strut was positioned like an exoskeleton on the anterior chest wall. Pragmatic aspects of this modification in the setting of combined surgical treatment are discussed and a systematic review of combined surgical aortic aneurysm repair and PEX correction is presented.

## 2. Case Description

**Figure 1:**
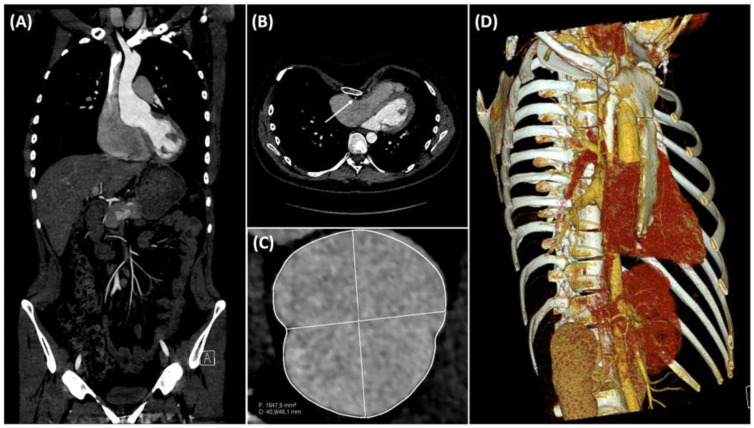
Preoperative electrocardiogram-gated computed tomography angiography (CTA); (**A**) frontal view of CTA, which revealed a pyriform annuloaortic aneurysm; (**B**) axial view of CTA thorax scan, showing displacement of mediastinal contents into the left hemithorax with consequent marked levocardia and cardiac constriction; the arrow indicates the immediate retrosternal course of the right coronary artery behind the sternum; (**C**) two-dimensional reconstruction of the computed tomography data set showing its asymmetric aneurysmal alternation; (**D**) three-dimensional reconstruction of the CTA data set showing compression of the right heart by the sternum.

An 18-year-old male patient with MS, known *FBN1* mutation, and congenital PEX presented with acute atypical retrosternal chest pain that radiated to the back and differed in character from his chronic chest pain. Electrocardiography (ECG) and laboratory testing ruled out acute myocardial infarction. One month earlier, the patient had been referred to our institution because an aortic root dilatation had increased by 6 to 8 mm over 6 months to a diameter of 53 mm on his most recent transthoracic echocardiography (TTE) and magnetic resonance angiography. The same studies showed a 47 mm fusiform aneurysm of the ascending aorta. At this point, a surgical therapy option was discussed with the patient due to the rapid size-progression of the aneurysm, and an elective inpatient admission was scheduled. ECG-gated computed tomography angiography (CTA) of the aorta excluded acute aortic dissection, confirming the previous findings (Figure 1A). The right coronary artery (RCA) had a direct retrosternal course, which was separated from the sternum only by the pericardium (Figure 1B). The patient’s PEX had resulted in decreased exercise tolerance. His symptoms correlated with the recent imaging findings, which revealed a Haller index of 4.5 and displacement of mediastinal contents into the left hemithorax with consequent marked levocardia and cardiac constriction (Figure 1B,D). Preoperative diagnostic imaging also indicated an asymmetric anatomy of the aortic root dilatation (Figure 1C).

TTE showed an impression of the right heart at the level of the tricuspid valve but competent systolic function of the right and left ventricles without diastolic dysfunction. No significant valve pathology was detected. Pulmonary function testing revealed mild restrictive lung disease. Preoperative routine laboratory values were unremarkable. Because of the large aortic root aneurysm, its acute and rapid enlargement in diameter in association with new acute pain symptoms, and severe PEX with a Haller index of 4.5, surgery was indicated. After a thorough multidisciplinary case evaluation, we elected to proceed with single-stage combined Bentall and modified Ravitch procedures and the patient consented. In this case, a mechanical Bentall procedure was planned due to the asymmetry of the aortic root and at the request of the well-read patient. Nonetheless, the type of procedure on the aortic valve was not limited by the planned simultaneous Ravitch correction. Therefore, in general, a valve-sparing technique would also be possible, depending on the aortic root pathology and wishes of the well-informed patient. The surgical procedure was performed in collaboration with pediatric surgery colleagues.

**Figure 2:**
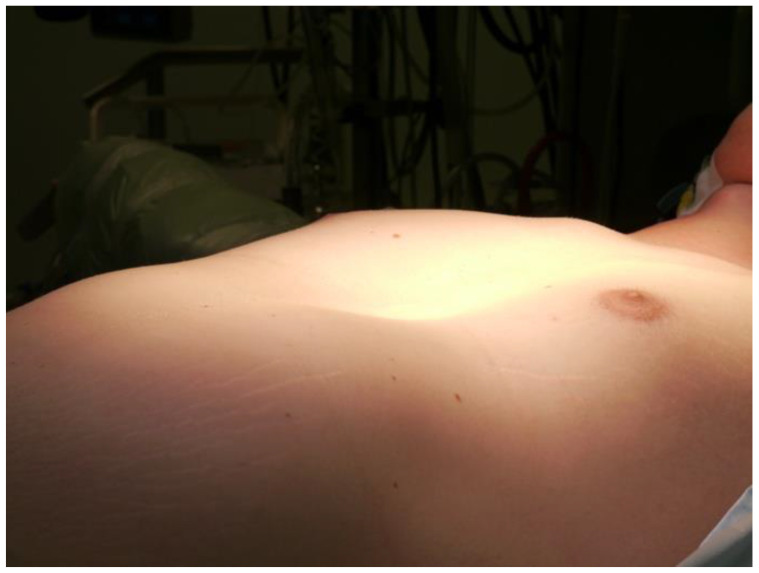
Photo from the intraoperative setting: patient in supine position; the pectus excavatum is clearly visible here.

After induction of general anesthesia, the patient was placed in the supine position (Figure 2) and a standard median sternotomy skin incision was made. Due to the close retrosternal course of the RCA, upward traction with Volkmann bone hook retractors was performed during the sternotomy. The conventional sternotomy spreader was placed and the pericardium was opened. Despite remarkable levocardia, cardiac exposure for the procedure was adequate. After systemic full heparinization (target activated clotted time, >400 s) the ascending aorta was cannulated, and venous drainage was instituted via insertion of a two-stage cannula into the right atrium to commence cardiopulmonary bypass (CPB). The left ventricular vent was placed via the right superior pulmonary vein and directed toward the left ventricle. The aorta was cross-clamped, and cardiac arrest was induced by antegrade application of Brettschneider cardioplegia through the aortic root. After opening the ascending aorta, the aortic valve was exposed to reveal a tri-leaflet unsalvageable valve, because the aortic root was extremely asymmetric and the leaflets were prolapsing, as revealed by preoperative diagnostic imaging. Under normothermic CPB, the Bentall procedure was performed—the aortic root and ascending aorta were replaced with a 23 mm mechanical valved conduit and the coronary ostium buttons were successively directly reimplanted in the aortic prosthesis [13]. Weaning and separation from CPB went smoothly. Substernal and pericardial chest tubes were placed. Protamine was administered followed by careful surgical hemostasis. The pericardium was closed. Fibrinogen was substituted (guided by thromboelastometry) to optimize humoral hemostasis during sternal osteosynthesis with stainless steel wires. Total CPB time was 56 min; aortic cross-clamp time was 44 min.

**Figure 3:**
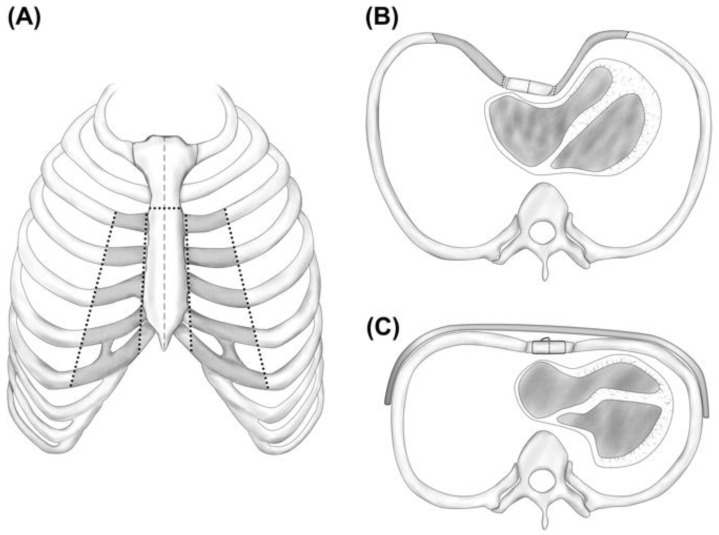
Schematic representation of the surgically corrected pectus excavatum (PEX); (**A**) the dashed line marks the median sternotomy; the oblique vertical dotted lines along the third to seventh rib indicate the bilateral costochondral osteotomies; the horizontal dotted line on the sternum implies the transverse anterior osteotomy; the gray-shaded rib areas signify the removed rib segments; (**B**) horizontal view of the performed bilateral costochondral osteotomies; note the left thoracic displacement and impression of the heart due to the prominent PEX; (**C**) the results of the surgically corrected PEX reinforced in its shape by a metal bar.

The modified Ravitch procedure was then performed to repair the PEX, starting with bilateral mobilization of the pectoralis muscles to the anterior axillary line (Figure 3). The muscles were lifted off the sternum with a mobilized rectus muscle flap to properly expose the sternum with the adjacent deformed costochondral cartilages so that bilateral costochondral osteotomies could be performed. The osteotomies were performed at the costosternal junction of the third through seventh ribs while preserving the perichondrium and both internal mammary arteries. A transverse anterior osteotomy of the sternum immediately above the third costal notch was performed while the sternal tabula interna was preserved (Figure 3A). A more lateral wedge-shaped subchondral costotomy was performed at the apex of the pathologic rib-bending of the third through seventh ribs (Figure 3B). Consequently, the ventrally elevated sternal segment was reinforced in its position with episternal placement of a 16-inch steel bar that had previously been bent to the intended convex thoracic shape. For this, a submammary longitudinal skin incision was made bilaterally through which the steel bar was guided at the height of the fourth rib through a submuscular tunnel over the osseous anterior chest wall like an exoskeleton (Figure 3C). The bar was secured bilaterally by polydioxanone sutures laced around the fourth ribs and fixed in position by lateral bar stabilizers. The mobilized sternal segment was also fixed to the bar while the remaining detached ribs were reattached to the sternum using polydioxanone cords. A chest tube was positioned in the right pleural cavity and two Redon drains were placed under the muscle flaps followed by layered closure of the wound. Before the patient was transferred to the intensive care unit, thromboelastometry was performed, which showed no abnormalities. Total duration of the modified Ravitch procedure was approximately 270 min.

**Figure 4:**
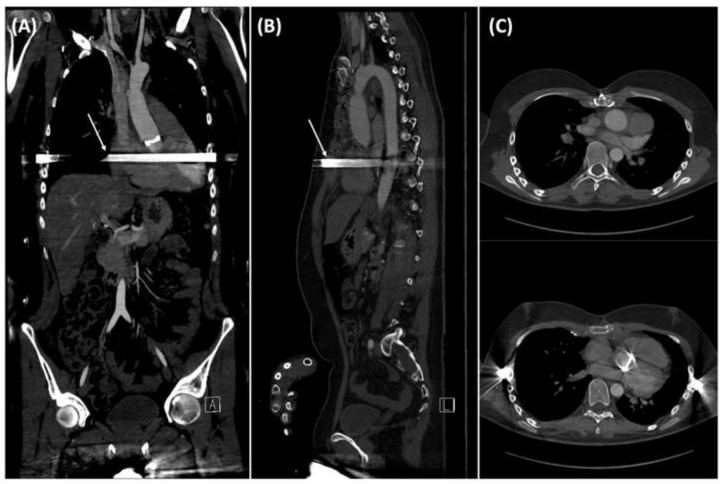
Postoperative electrocardiogram-gated computed tomography angiography (CTA) before discharge; (**A**,**B**) frontal and sagittal view of postoperative CTA showing a regular fit and course of the valved aortic conduit; the arrows indicate the episternal position of the metal bar; (**C**) axial views of CTA reveal the corrected pectus excavatum and resulting decompression of the right ventricle and mesocardia.

**Figure 5:**
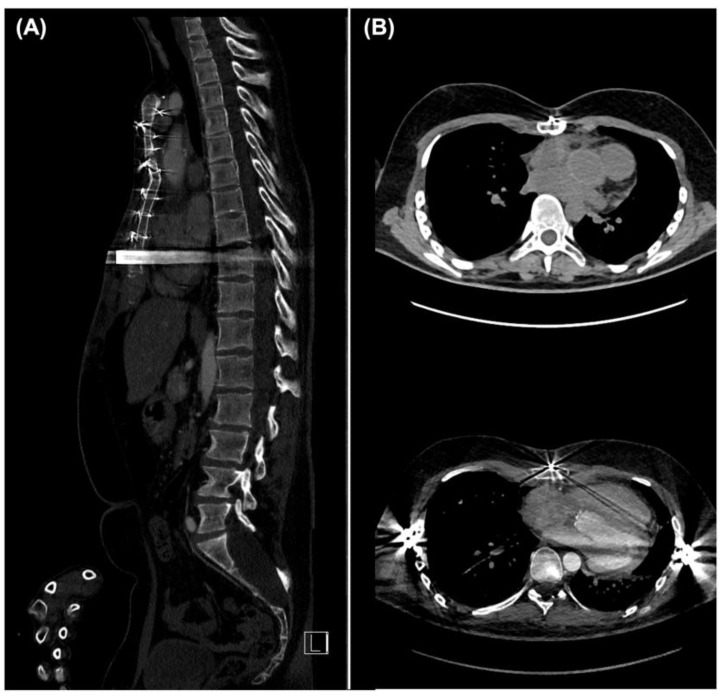
Postoperative electrocardiogram-gated computed tomography angiography (CTA) follow-up examination after three years; (**A**) sagittal and (**B**) axial views of postoperative CTA showing an adequate bony coalescence of the anterior chest wall without residual or recurrent funnel chest.

The patient remained hemodynamically stable after surgery, and he was extubated on the first postoperative day. Postoperative echocardiography revealed a right pleural and circular pericardial effusion. On the eighth postoperative day, a chest tube was inserted and pericardiocentesis was performed because of effusion progression. The remainder of his inpatient stay proceeded uneventfully. The patient was very satisfied with the cosmetic outcome of surgery. Postoperative ECG-guided CTA of the aorta (Figure 4) showed a regular fit and course of the valved aortic conduit (Figure 4A,B) and mesocardia (Figure 4C). TTE revealed proper valvular prosthesis function. The patient presented to our institution for annual follow-ups. The aortic valve prosthesis continued to demonstrate appropriate function at the annual TTE follow-up visits. At the end of three years, a repeat CTA examination was performed which showed adequate bony coalescence of the reconstructed anterior chest wall without residual or recurrent funnel chest (Figure 5A,B). Thus, the decision was made to remove the pectus bar, which was removed after three years without any periprocedural complications.

## 3. Case-Based Discussion

The Ravitch sternochondroplasty, first described in 1949, has undergone numerous modifications over the years. In the modification described here, the steel strut was positioned over the sternum like an exoskeleton on the anterior chest wall. In a setting of combined surgery via midsternotomy, this technique is convenient. First, episternal implementation might lower the risk of sternal dehiscence, as the impact of tensile forces from a substernal steel bar are avoided. Removal of the steel bar, which eventually must be performed, is easier and probably less risky because pericardial and other intrathoracic adhesions are not a factor. Moreover, pericardiocentesis can be performed if necessary because the steel bar does not impede substernal access. Furthermore, the bar was secured bilaterally by absorbable polydioxanone sutures laced around the fourth ribs. The nonabsorbable sutures were deliberately avoided to prevent fistula formation to intrathoracic organs along the sutures to the pectus bar. This was to facilitate subsequent removal of the pectus bar.

Our patient had PEX-specific complaints reflected in imaging and functional findings. Because severe pectus excavatum deformity may jeopardize postoperative cardiac function as a result of cardiac compartment syndrome and myocardial edema after CPB and cardioplegic arrest, we elected to proceed with single-stage surgery to avoid compromising the success of aortic aneurysm repair. The Haller index of 4.5 contributed to our decision-making process. To determine the Haller index, the transverse diameter of the rib cage is measured at the level of the smallest distance between the anterior surface of the vertebral body and the posterior surface of the sternum and divided by the axial diameter [14]. A Haller index ≥ 3.25 is considered an indication for surgical treatment.

Conventional midline complete sternotomy was performed to gain adequate control of the aortic root, entire ascending aorta, and supra-aortic branches. Once median sternotomy has been selected, a vertical skin incision is inevitable, even if a horizontal skin incision would be preferable to minimize scarring in the “keloid triangle.”

To counteract the increased risk of bleeding, the Ravitch procedure was initiated after sternal closure, heparin antagonization, and thromboelastometry-based substitution of coagulating agents. Blood loss was also minimized by preserving the perichondral sheets to save the intercostal and internal mammary vascular bundles, the latter of which minimizes the risk of sternal dehiscence.

A pectus bar was used to achieve early stabilization of the chest wall and avoid compromising the cosmetic result. In our facility, as is also recommended in the literature, the pectus bar is removed after three years [15]. CTA examination excluded hyperossification along the pectus bar and fistula formation or adhesions of intrathoracic organs to it. Since the pectus bar was episternally implemented, its removal was without complications, so that the patient could be discharged the day after the surgical procedure.

## 4. Systematic Review of Single-Stage Bentall and Ravitch’s Surgical PEX Correction

### 4.1. Introduction

Cardiovascular lesions and thoracic skeletal deformities are frequent complications in MS patients [16]. However, the need to perform cardiac surgery in combination with surgical correction of pectus deformity is rare [6], which is why only solitary case reports or small case series regarding the topic have been published. Because analyzing two-stage surgeries and other combined surgeries resulted in a wide heterogeneity of treatment approaches [17], we reviewed only case reports or series that exclusively presented combined Bentall and modified Ravitch procedures. Our aim was to discuss previously described surgical treatment strategies and assess their techniques and early outcomes.

### 4.2. Patients and Methods

We conducted a systematic review of the scientific literature according to the Preferred Reporting Items for Systematic Reviews and Meta-Analyses [18]. Two independent reviewers (Asen Petrov and Ali Taghizadeh Waghefi) searched English-language peer-reviewed articles in PubMed and Google Scholar published before or on 31 December 2021 using the following search terms: “Marfan syndrome”, “pectus excavatum”, “funnel chest”, “repair of funnel chest”, “aortic root dilatation”, “aortic root aneurysm”, “aortic aneurysm”, “Bentall surgery”, “composite graft”, “pectus correction”, “Ravitch procedure”, and “modified Ravitch procedure”. The PubMed “related articles” function was added to broaden the search options. The reference lists of all retrieved articles were checked for additional potentially relevant publications. The Cochrane Library was also searched for reviews with the same or similar subject matter. Study-selection criteria included publications reporting combined Bentall and modified Ravitch procedures in MS patients of any age group. Non-English articles were omitted. Subsequently, after removal of duplicates, a manual search was performed to screen articles for eligibility based on the title, abstract, and full text. Publications reporting two-stage surgery or cardiosurgical interventions other than or in addition to the combined Bentall and modified Ravitch procedures or patients with forms of inherited connective tissue disease other than MS were excluded. All relevant articles were imported into the reference management software EndNote 20. A manual search was then performed to verify articles’ eligibility. The reviewers also independently extracted data from each appropriate study and entered them into an electronic record. The extracted data comprised the first author’s surname, year of publication, dates of the study, type of the study (case report/case series), sample size, data regarding comorbidities, clinical presentation, Haller index, surgical strategy, surgical access, and patient outcome. Any differences were resolved by discussion and reaching a consensus. Because of the small number of patients and non-random selection of the patient cohort, the data were insufficient for an analytical comparison, so only a descriptive analysis was performed. Categorical variables were expressed as numbers and percentages, and continuous variables were expressed as means and standard deviations. Data for missing values were not imputed. R software (version 4.03) was used to perform all analyses.

### 4.3. Results

**Figure 6:**
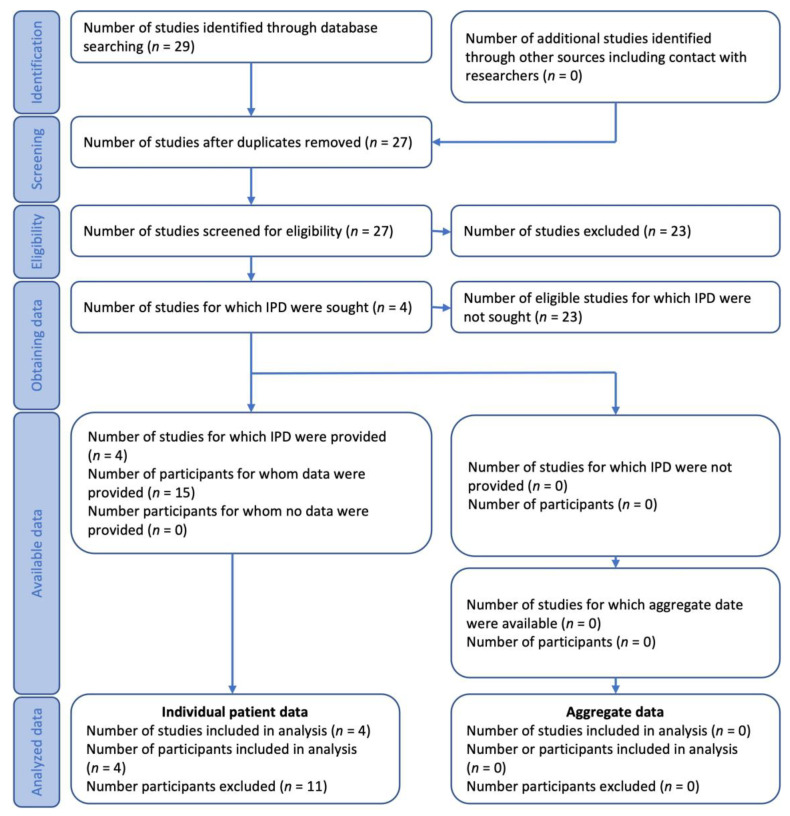
PRISMA flow-chart diagram summarizing the method of cases for the study; IPD, individual patient data.

The systematic search yielded a total of 29 peer-reviewed publications; 23 did not meet the selection criteria and were excluded. Reasons for exclusion were as follows: cardiac procedure performed in addition to Bentall procedure (*n* = 6), language other than English (*n* = 5), PEX-correction technique other than modified Ravitch procedure (*n* = 5), valve-sparing ascending aortic replacement (*n* = 3), no correction of PEX (*n* = 3), and staged surgery (*n* = 1). The remaining six articles were adjusted for duplicates, resulting in a total of four eligible papers, three of which were case reports and one of which enclosed a patient of a case series (Figure 6) [19,20,21,22]. Furthermore, the patient presented in this report was added. Table 1 summarizes the essential characteristics of the five analyzed patients and their surgical details. Patient age ranged from 14 to 28 years with a median of 22.5 years and a mean of 22 ± 5.9 years. Three patients were male. The Haller index was not reported in any article (the Haller index in our patient was 4.5). All case studies indicated a midline vertical skin incision over the sternum. The most common surgical approach to the mediastinum was midsternotomy (*n* = 4); one article reported left-sided costotomy. Only a single article reported the cannulation technique for connection to CPB (aorto-bicaval cannulation) [22]; aorto-right-atrial cannulation was performed in our patient. In one article, an emergency modified Ravitch procedure was performed because of hemodynamic instability after sternal occlusion caused by sternal compression of the right ventricle [21]. In the remaining publications, PEX correction was a planned elective procedure. One case report described combined emergency Bentall surgery and modified PEX correction using the Adkins and Blades technique (a modification of the Ravitch procedure) in an MS patient with acute type A aortic dissection (classified by Stanford); left-sided costotomy was used for surgical access [20]. The Bentall procedure was elective in the remaining articles. The CPB time (mean 126.5 ± 48.4 min, median 142 min) and aortic cross-clamp time (mean 80.5 ± 25.7 min, median 89.5 min) were reported in three patients, and were supplemented with our patient’s data [20,21,22]. Likewise, the duration of the Ravitch procedure was reported in three patients; mean and median times (including our patient) were 209.3 ± 74.9 min and 232.5 min, respectively [19,20,21]. The total surgical time was available in three cases [19,20,21]; mean and median times (including our patient) were 394.5 ± 80.4 min and 365 min, respectively. Thus, PEX correction accounted for approximately 53% of the total surgical time. In all cases, a metal bar was used to reinforce the pectus in its corrected shape. No serious or persistent postoperative morbidity was reported, and postoperative mortality was in all cases zero.

### 4.4. Discussion

Cardiovascular lesions and thoracic skeletal deformities are frequent complications in patients with MS [16]. The need to perform cardiac surgery in combination with correction of pectus deformity is rare [6]. Reports of combined Bentall and Ravitch procedures are particularly rare. Our review of the literature yielded only four previous cases of single-stage combined Bentall and Ravitch surgery.

The main question is whether and how patients with concomitant pectus deformity and cardiovascular disease should be treated surgically in one step. Currently, there is no consensus. A few publications advocate staged repair because of concerns about serious complications such as limited surgical exposure, severe bleeding, and prolonged operative time [16,23,24,25]. Conversely, most recent case reports and case series consider single-stage surgery to be safe. Concomitant surgical correction of chest wall deformities may even be obligatory, as severe PEΧ deformity may compromise postoperative cardiac function. The major advantages of a single-stage procedure are immediate cosmetic satisfaction and avoidance of cardiac compression after thorax closure. However, the risks of such a combined procedure cannot be neglected; these include the complexity of the procedure and the long duration of the operation. These factors may be associated with a higher rate of blood loss and consecutive transfusions [16,23,24,25,26]. The main advantage of staged surgery is the shorter operative time. Nonetheless, an uncorrected PEX may lead to impaired cardiovascular and respiratory function or increased intraoperative cardiac compression after thorax closure [21,27]. It should also be kept in mind that the second surgical procedure to correct PEX may be technically more challenging due to the adhesions [17]. In summary, it can be deduced from the literature that there has been a paradigm shift toward single-stage combination procedures over the years as surgical techniques have evolved.

Once the decision has been made to proceed with combined surgery, the key question of thoracic access technique arises. In addition to median sternotomy with transverse sternal split and median sternotomy with staged bilateral costochondral osteotomy both before and after the surgical procedure, parasternal approaches and sternal turnover techniques have been described. Thus, controversy persists regarding the best surgical approach. However, median sternotomy was the most commonly used surgical approach in previous publications. Adequate surgical-field exposure was reported in all cases.

Another point of contention is whether a vertical or horizontal skin incision should be performed for a satisfactory cosmetic result, as some authors consider a vertical incision in the keloid triangle to be cosmetically questionable. Nevertheless, our review showed that a longitudinal midline skin incision was used in all patients who underwent single-stage Bentall and modified Ravitch surgery. Postoperative keloid formation was not reported in any case.

Since its initial description in 1949, the Ravitch sternochondroplasty has undergone numerous changes and modifications over the years to allow simultaneous surgical treatment of cardiac lesions. In this respect, controversy remains over whether the use of a metal strut is essential to reinforce the pectus in its surgically corrected shape. Some authors have expressed concern that a metal strut may be associated with complications such as migration or dislocation, which may cause intrathoracic organ injury [19]. However, other studies have reported superior surgical pectus correction when using temporary metal rods because the rods provide immediate stabilization of the chest wall which causes less pain [28]. Our review found that the use of temporary metal bars achieved satisfactory cosmetic results and was not associated with any complications.

### 4.5. Conclusions

Single-stage combined Bentall and modified Ravitch surgery is an underutilized surgical approach. Its use in MS patients with concomitant PEX and ascending aortic aneurysm that require surgical treatment warrants further investigation. Midsternotomy seems to be a viable access route that provides sufficient exposure in the single-stage surgical setting. Although operative time is long, the intraoperative and postoperative risks appear to be low and manageable.

**Table 1:** Main data of all included patients in this review undergoing combined Bentall and modified Ravitch procedure.

Author, Year	Age	Sex	HI	Skin Incision	Access	Cannulation	Aortic Surgery	PEX Surgery	Complications	CPB T(min)	XCT (min)	Duration of Ravitch (min)	TST (min)
Okay et al., 2008 [19]	27	M	N/A	Longitudinal midline	Midsternotomy	N/A	Bentall	Modified Ravitch	-	-	-	102	335
Schwill et al., 2010 [20]	28	F	N/A	Longitudinal midline	Left-sided costotomy	N/A	Bentall	Modified Ravitch	-	166	98	249	513
Stephens et al., 2012 [24]	23	F	N/A	Longitudinal midline	Midsternotomy	N/A	Bentall	Modified Ravitch	Emergent Ravitch	140	81	216	360
Kansara et al., 2013 [22]	14	M	N/A	Longitudinal midline	Midsternotomy	Aorto-bicaval	Bentall	Modified Ravitch	-	144	99	N/A	N/A
Case report	18	M	4.5	Longitudinal midline	Midsternotomy	Aorta and right atrium	Bentall	Modified Ravitch	Pericardial and pleural effusion	56	44	270	370

Abbreviations: M, male; F, female; HI, Haller index; N/A, not available; CPBT, cardiopulmonary bypass time; XCT, aortic cross clamp time; PEX, pectus excavatum; TST, total surgery time.

## Data Availability

Detailed data presented in the study are available on request from the corresponding author.
